# Subtype classification of malignant lymphoma using immunohistochemical staining pattern

**DOI:** 10.1007/s11548-021-02549-0

**Published:** 2022-02-11

**Authors:** Noriaki Hashimoto, Kaho Ko, Tatsuya Yokota, Kei Kohno, Masato Nakaguro, Shigeo Nakamura, Ichiro Takeuchi, Hidekata Hontani

**Affiliations:** 1 Center for Advanced Intelligence Project, RIKEN, 1-4-1 Nihonbashi, Chuo-ku, Tokyo, 103-0027 Japan; 2 Department of Computer Science, Nagoya Institute of Technology, Gokiso-cho, Showa-ku, Nagoya, 466-8555 Japan; 3 Department of Pathology and Laboratory Medicine, Nagoya University Hospital, 65 Tsurumai-cho, Showa-ku, Nagoya, 466-8560 Japan; 4 Department of Pathology, Kurume University School of Medicine, 67 Asahi-machi, Kurume, 830-0011 Japan; 5 Department of Pathology and Laboratory Medicine, Nagoya University Graduate School of Medicine, 65 Tsurumai-cho, Showa-ku, Nagoya, 466-8560 Japan

**Keywords:** Digital pathology, Image classification, Typicality, Malignant lymphoma, Instance selection

## Abstract

**Purpose:**

For the image classification problem, the construction of appropriate training data is important for improving the generalization ability of the classifier in particular when the size of the training data is small. We propose a method that quantitatively evaluates the typicality of a hematoxylin-and-eosin (H&E)-stained tissue slide from a set of immunohistochemical (IHC) stains and applies the *typicality* to instance selection for the construction of classifiers that predict the subtype of malignant lymphoma to improve the generalization ability.

**Methods:**

We define the typicality of the H&E-stained tissue slides by the ratio of the probability density of the IHC staining patterns on low-dimensional embedded space. Employing a multiple-instance-learning-based convolutional neural network for the construction of the subtype classifier without the annotations indicating cancerous regions in whole slide images, we select the training data by referring to the evaluated typicality to improve the generalization ability. We demonstrate the effectiveness of the instance selection based on the proposed typicality in a three-class subtype classification of 262 malignant lymphoma cases.

**Results:**

In the experiment, we confirmed that the subtypes of typical instances could be predicted more accurately than those of atypical instances. Furthermore, it was confirmed that instance selection for the training data based on the proposed typicality improved the generalization ability of the classifier, wherein the classification accuracy was improved from 0.664 to 0.683 compared with the baseline method when the training data was constructed focusing on typical instances.

**Conclusion:**

The experimental results showed that the typicality of the H&E-stained tissue slides computed from IHC staining patterns is useful as a criterion for instance selection to enhance the generalization ability, and this typicality could be employed for instance selection under some practical limitations.

## Introduction

Malignant lymphomas have more than 70 subtypes, and pathologists are required to identify the subtype from a set of microscopic images of a specimen that is invasively extracted from a patient to determine the treatment of the patient [1]. In a pathological diagnosis of malignant lymphoma, a hematoxylin-and-eosin (H&E)-stained tissue slide is first observed to infer the candidate subtypes, and a set of immunohistochemical (IHC) stains that are required to identify the subtype is then selected. The subtype is finally identified by observing the tissue slides stained with the selected IHC stains and by considering their expression patterns. Currently, with the widespread use of whole slide images (WSIs) and the development of machine learning techniques, the image analysis of digital pathology has been accelerated, and there have been studies conducted on image classification [2–4], detection [5–7], segmentation [8–10], and survival prediction [11, 12]. The subtype classification of digital pathological images would be helpful in practical diagnoses as a computer-aided diagnosis application that can provide pathologists with a second opinion. The objective of this study is to construct a classifier that can predict subtypes for a given WSI of an H&E-stained tissue specimen of malignant lymphoma.

For the construction of the subtype classifier, we use a set of WSIs of H&E-stained tissue specimens with slide-level subtype labels. The generalization ability of the classification model depends on the training data, and the constructed classifier often overfits the training data such that the generalization ability is degraded when the size of the training data is small. To improve the generalization ability while using a limited number of training data, it is necessary to construct appropriate training data. In the context of instance selection, it is known that the removal of atypical instances from the training data of a classification model can enhance the generalization ability [13, 14]. Even in the subtype classification of pathological images, it is assumed that an instance, whose H&E-stained tissue image shows the atypical appearance, can degrade the generalization ability of the classification model. In our classification problem that predicts subtypes for a WSI of an H&E-stained tissue slide, each *case* corresponds to *instance* in the context of instance selection. If the criterion indicating how typical the H&E-stained tissue slide of each instance is known, the instance selection can be applied to the training data based on such typicality measures. In this paper, we propose a method that selects appropriate training data based on the typicality of the H&E-stained tissue slides of malignant lymphomas.

In general, a database for digital pathology manages information of each instance as a pair of a WSI and the corresponding medical record wherein patient metadata, the set of names of IHC stains used for the diagnosis, the identified subtype, and other findings are described by pathologists. Although these medical records include no information about the typicality of the morphological features in observing H&E-stained tissue slides, the set of IHC stains used for the definitive diagnosis is determined based on the candidates of the subtypes that are inferred by the first observation of the H&E-stained tissue slide. We note that the same set of IHC stains is not always used to diagnose instances wherein the definitive diagnosis is of the same subtype. A specific set of IHC stains is commonly used in almost all instances wherein the definitive diagnosis is of the same subtype; however, some additional IHC stains are used in many of the instances. Herein, we hypothesize that the morphological features observed in the H&E-stained tissue slides are typical for the corresponding subtype when only the set of IHC stains that is common among the instances of the same subtype is used for the definitive diagnosis. When the morphological features are typical for the subtype and hence only the subtype is inferred as the candidate at the first observation of the H&E-stained slide image, the pathologist then selects only the optimal set of IHC stains that are required to confirm that the candidate subtype is correct. In contrast, if the morphological features of the H&E-stained tissue slide are atypical for the subtype, and hence, multiple subtypes are inferred as candidates at the first observation, additional IHC stains are required to identify the subtype for the definitive diagnosis. Based on this hypothesis, we quantitatively evaluate how typical the H&E-stained tissue slide is by the set of IHC stains used in the diagnosis and employ the evaluated typicality as the criterion for the instance selection of the training data.

In this paper, we propose a method that quantitatively evaluates the typicality of the H&E-stained tissue slides and selects the training data for the construction of subtype classifiers by referring to the evaluated typicality in order to improve the generalization ability. We demonstrate that instance selection based on the proposed typicality improves the generalization ability of the subtype classification model by a three-class classification experiment with 262 cases of malignant lymphoma. In the experiment, WSIs of the H&E-stained tissue sections were used, wherein a partial region of the tissue specimen was cancerous, and the WSIs had no pathologist annotation indicating cancerous regions. We employed a multiple-instance-learning-based (MIL-based) convolutional neural network (CNN) that could automatically focus on image patches of cancerous portions from bags that comprised sets of image patches extracted from the entire tissue slides, and the proposed method provided the criteria for selecting the appropriate training data for the MIL-based subtype classification.

The contributions of this paper are as follows:

- We proposed a method for evaluating the typicality of H&E-stained tissue slides for the criteria in the instance selection, which was derived from the sets of IHC stains used in the pathological diagnosis of malignant lymphomas.
- We analyzed the sets of IHC stains in the dataset consisting of clinical lymphoma cases and revealed the relationship between the subtypes and the sets of IHC stains, wherein we could observe similar sets of IHC stains between different subtypes and various sets of IHC stains within a subtype.
- We demonstrated an MIL-based CNN for classifying three types of malignant lymphoma tissues, of diffuse large B cell lymphoma (DLBCL), angioimmunoblastic T-cell lymphoma (AITL), and classical Hodgkin’s lymphoma (CHL) to confirm the effectiveness of the typicality-based instance selection.

## Preliminaries

### Related works

*Malignant lymphoma in digital pathology*

Malignant lymphomas comprise a group of blood cancers that develop from lymphocytes—a type of white blood cell [1]—and their pathological diagnosis is complicated. It is known that predicting the subtypes of lymphomas from an H&E-stained tissue slide is quite difficult even for expert pathologists because of the various subtypes and the diversity in the subtype-specific appearance in tissue specimens of malignant lymphomas. To the best of our knowledge, there have been only a few studies on the subtype classification of malignant lymphomas, which is in contrast to the several studies conducted on the subtype classification of other diseases [2–4]. The literature [15] reported the automated image classification of B cell lymphomas, wherein the classifier was trained for benign, DLBCL, Burkitt lymphoma, and small lymphocytic lymphoma using a set of annotated image patches. Miyoshi [16] performed the classification of DLBCL, follicular lymphoma, and reactive lymphoid hyperplasia with patch-level annotated images. In both studies, high classification performances were realized for lymphoma subtypes, but the problem settings were relatively simple, wherein the image patches were extracted from an entire WSI based on the pathologist’s annotation. A WSI, which is a digitized pathological image of the entire glass slide, is large (approximately 100,000$\times$100,000 pixels), and in general, only some regions in the image are cancerous. Although the image classification has to be performed based on cancerous regions that have class-specific features, it is prohibitively time-consuming to annotate the cancerous regions in many images. In the case of clinical applications, it is expected that the subtype classification of the pathological images is performed on WSIs that have no annotations for cancerous regions. We employ an MIL technique to classify WSIs as weakly annotated images, and the explanation of the MIL-based classification is described in the next section.

*Multiple instance learning* In cases without a pathologist’s annotation of the cancerous regions in the WSIs, MIL techniques have demonstrated excellent performance in pathological image analyses [17–22]. MIL-based classification conducts label prediction on each bag, which includes multiple image patches sampled from the WSI. The label provided for each bag denotes the subtype of the WSI from that image patches of the bag are sampled. In binary classification, a positive bag that is generated from a positive-class WSI has at least one positive image patch, and a negative bag that is generated from a negative-class WSI only has negative image patches. The subtype identification in this study is a problem of multi-class classification, and it is assumed that each bag has at least one image patch that has class-specific features. Using MIL, we can construct a subtype classifier that can automatically select image patches in the bag that contribute to subtype identification.

Several MIL methods for aggregating features of the image patches in a bag into the bag’s features have been proposed [19, 23]. We employ an attention-based method that can obtain explainable results and visualize tumor-specific regions in the WSI [19]. The attention-based MIL has also been applied to the subtype classification of malignant lymphomas, and a better result was reported for discriminating DLBCLs [24]. They performed a two-class classification of malignant lymphomas [24], and a more complicated problem setting was expected in clinical applications. When the MIL-based techniques are applied to multi-class classification, the selection of image features that contribute to classification is more difficult, and atypical instances would degrade the generalization ability more strongly owing to the heterogeneity of morphological features in the H&E-stained tissue slides. The instance selection for the training data is therefore more important in our problem setting.

### Problem setup

Let *N* be the number of instances of malignant lymphoma, and we define $[N]:=\{ 1,\dots ,N \}$. The lymphoma dataset is represented as ${\mathcal {T}}=\{({\mathbb {X}}_n, {\mathbb {Y}}_n,{\mathbb {S}}_n)\}_{n=1}^N$, where ${\mathbb {X}}_n$ is a WSI for a patient, and $n \in [N]$. Each patient’s data has two types of labels corresponding to ${\mathbb {X}}_n$: ${\mathbb {Y}}_n$ is a *K*-dimensional one-hot vector for the multi-class label of *K* subtypes, and ${\mathbb {S}}_n$ is an *L*-dimensional multi-label binary vector that represents the IHC staining patterns, i.e., the combination of IHC stains used for the definitive diagnosis. In a medical record of malignant lymphomas, a list of IHC stains used in the definitive diagnosis is provided for each instance, and their expression patterns are described in the form of positive or negative. Each element of ${\mathbb {S}}_n$ corresponds to a different IHC stain, where a value of 1 indicates that the corresponding IHC stains were used in the diagnosis, while 0 indicates that they were not used. For example, we consider the case wherein we use three types of IHC stains CD20, CD3, and CD30 (in this example, $L=3$), and each element of the multi-label vector ${\mathbb {S}}_n$ corresponds to [CD20, CD3, CD30]. Here, if a medical record for an *n*-th patient indicates that CD20 and CD30 were used in the diagnosis, the multi-label vector ${\mathbb {S}}_n$ is given as [1, 0, 1]. In our experimental dataset, 87 types of IHC stains are originally considered as candidates and 9.84 IHC stains are used for an instance on average.

Our classification problem is to predict the subtype label ${\mathbb {Y}}_n$ for an input WSI ${\mathbb {X}}_n$. To test our hypothesis, we compute the subtype typicality of each H&E-stained tissue slide from the corresponding IHC staining pattern and perform a classification experiment with instance selection based on the calculated typicality. It should be noted that ${\mathbb {S}}_n$ is used only for calculating typicality and instance selection, and the subtype is predicted from only WSIs ${\mathbb {X}}_n$ using the MIL classification model. A set of indices of the bags obtained from the *n*-th WSI are denoted by ${\mathcal {B}}_n$, and the set of image patches in the *b*-th bag ($b \in {\mathcal {B}}_n$) is denoted by $\{ \varvec{x}_i \} _{i \in {\mathcal {I}}_b}$, where ${\mathcal {I}}_b$ denotes a set of indices of the image patches.

## Methods

Our proposed method improves the generalization ability of the subtype classifier by selecting instances that have a *typical* appearance for each subtype and using them for training. In this section, we explain the definition of subtype typicality and instance selection using subtype typicality. An MIL-based CNN model for classifying the subtypes of malignant lymphoma is also described.

### Typicality of subtypes

The proposed method evaluates the typicality of the H&E-stained tissue slides using the similarity of IHC staining patterns used in the definitive diagnosis. Although there are differences in usage frequency for the pathological diagnoses of malignant lymphomas, more than 100 IHC antibodies are considered as candidates for the IHC stains. A set of IHC stains can have a variety of instances even if the subtypes of their malignant lymphomas are the same because H&E-stained tissue slides have various appearances even among the same subtype. If the subtypes can be identified with high confidence using only H&E-stained WSIs, then only the IHC stains that are common in each subtype would be used. In contrast, if there is confusion in inferring the subtypes from the H&E-stained WSIs, all the IHC stains that are required for the identification of each of the candidate subtypes are used. It is assumed that IHC staining patterns described in medical records latently have the typicality of the appearance of H&E-stained WSIs. The proposed method, therefore, quantitatively evaluates how typical the appearance of H&E-stained WSIs is for each subtype by referring to a set of IHC stains used for realizing a definitive diagnosis.

First, we define the distance between two sets of IHC stains, and all the IHC staining patterns ${\mathbb {S}}_n$ in the data ${\mathcal {T}}$ are embedded in a low-dimensional space via multi-dimensional scaling (MDS) [25] based on the defined distance. It should be noted that no information of the subtypes is used for the embedding, although each embedded instance has a label of the subtype. The probability density distribution of IHC staining patterns in the embedded space is then estimated for each subtype using the embedded data that have the corresponding subtype label. The typicality of each instance is defined by the ratio of the probability density of the corresponding subtype to those of all subtypes: a *typical* instance has a higher probability density for the corresponding subtype without overlapping of the probability density of the other subtypes, while an *atypical* instance has the overlapping of probability density for the multiple subtypes.

The Hamming distance $d({\mathbb {S}}_m, {\mathbb {S}}_n)$, where $m\ne n \in [N]$, is used to measure the distance between the two IHC staining patterns ${\mathbb {S}}_m$ and ${\mathbb {S}}_n$:

1 $$\begin{aligned} d({\mathbb {S}}_m, {\mathbb {S}}_n) = \Vert {\mathbb {S}}_m - {\mathbb {S}}_n \Vert _1. \end{aligned}$$

Let $\varvec{u}_n \in {\mathbb {R}}^M$ ($M \ll L$) denote the data embedded by MDS, where $\{\varvec{u}_1, \varvec{u}_2, \dots , \varvec{u}_N \} = f_\mathrm{MDS}({\mathbb {S}}_1, {\mathbb {S}}_2, \dots , {\mathbb {S}}_N ; \varvec{D}),$ where $\varvec{D}$ denotes an $N\times N$ distance matrix, of which the (*m*, *n*) component is $d({\mathbb {S}}_m, {\mathbb {S}}_n)$, and $f_\mathrm{MDS}$ denotes a function that maps *L*-dimensional vectors that represent the IHC staining patterns in the *M*-dimensional space based on the distances in order to make it easier to estimate probability density distribution of IHC stains. Because MDS embeds the input feature vectors into low-dimensional space while preserving the relationship of the distances among instances, instances having similar IHC stains become close to each other even in the embedded space.

We employ kernel density estimation [26] to estimate the probability density distributions of embedded IHC staining patterns. The probability density distribution for the *k*-th subtype is computed from $\{ \varvec{u}_j\} _{j \in \mathcal J_k}$, where ${\mathcal {J}}_k$ is a set of indices such that $\mathcal J_k = \{ j \mid ({\mathbb {Y}}_j)_k = 1\}$, where $({\mathbb {Y}}_j)_k$ denotes the *k*-th component of the vector ${\mathbb {Y}}_j$. A kernel density estimator for subtype *k* is represented as:

2 $$\begin{aligned} {\hat{f}}_{k}\left( \varvec{u}_n \right) = \frac{1}{{|}{\mathcal {J}}_k{|} w} \sum _{i \in {\mathcal {J}}_k} G \left( \frac{ \varvec{u}_n - \varvec{u}_i}{w} \right) , \end{aligned}$$

where *G* is a Gaussian kernel function, and *w* is a bandwidth parameter. When $\varvec{u}_n$ is given as the embedded IHC staining pattern of the *n*-th instance, its typicality $t_{k}(\varvec{u}_n)$ for subtype *k* is calculated as the ratio of probability density for subtype *k* to probability densities of all subtypes:

3 $$\begin{aligned} t_{k}(\varvec{u}_n) = \frac{{\hat{f}}_{k}(\varvec{u}_n)}{\sum _{k=1}^{K}{\hat{f}}_{k}(\varvec{u}_n)}. \end{aligned}$$

In our experiment, instance selection is performed for the training and testing data based on the calculated typicality.

*Instance selection*

The typicality that we defined can be considered as the difficulty measure of inferring subtype candidates only from the H&E-stained tissue slide in the diagnosis. Typical instances would have the classifiable subtype-specific features in the H&E-stained WSIs because the pathologist could accurately infer the candidate subtype. This quantitative measure can be employed as a typicality criterion for instance selection. As mentioned in Section 1, if we know how typical each instance is, we can apply instance selection to a pathological image dataset to improve the generalization ability of the subtype classification, in which instances having the atypical appearance of H&E-stained WSIs are removed from the training data [13, 14]. In this paper, the calculation of typicality from the sets of IHC stains is the main novelty. Thus, we employ a simple method for the instance selection using the proposed typicality, wherein we change which typicality is focused and change the ratio of the typical and atypical instances in the training data. The details of instance selection are explained in the experimental setting.

### Attention-based MIL

In our classification experiment, we employ an attention-based MIL-CNN as the classification model [19, 24]. The attention-based MIL-CNN is known to be successful for unannotated WSIs, and it was also shown in the subtype classification of malignant lymphoma. In the subtype classification, the subtype of each patient is identified based on the WSI of the H&E-stained tissue slide by aggregating the predicted class labels of the bags obtained from the WSI. Specifically, given a WSI ${\mathbb {X}}_n$, the class label probability is simply predicted as $P({\hat{Y}}_n = k \mid {\mathbb {X}}_n) = p_k/\sum _{i \in K} p_i$, where

4 $$\begin{aligned} p_k = \exp \left( \frac{1}{{|}{\mathcal {B}}_n{|}} \sum _{b \in {\mathcal {B}}_n} \log P({\hat{Y}}_{b} = k) \right) . \end{aligned}$$

**Fig. 1:**
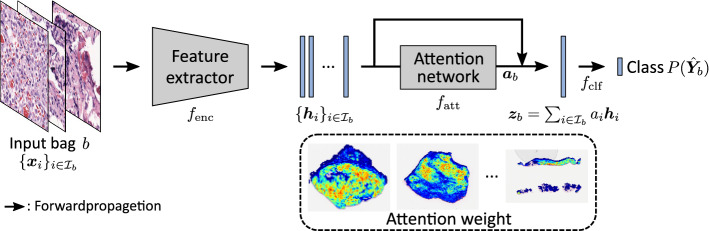
The illustration of the attention-based MIL-CNN used in our classification experiment. In the model, training is performed using a bag as an input, where an attention network can automatically compute the contribution of each image patch to the classification

Here, $P({\hat{Y}}_b = k)$ are the class label probabilities of bags $b \in {\mathcal {B}}_n$ for subtype *k*. Figure 1 illustrates the structure of our classification network, which consists of the three components. A feature extractor $f_\mathrm{{enc}}: \varvec{x} \mapsto \varvec{h}$ is a CNN that maps a $224 \times 224$-pixel image patch $\varvec{x}$ into a *Q*-dimensional feature vector $\varvec{h}$. An attention network $f_\mathrm{{att}}: \varvec{h} \mapsto a$ is a simple multilayer perceptron that outputs the attention weight of an input feature vector $\varvec{h}$. Feature vectors in a bag *b* are aggregated as a weighted sum, and the feature vector is obtained as $\varvec{z} = \sum _{i \in {\mathcal {I}}_b} a_i \varvec{h}_i$. A fully connected layer $f_\mathrm{{clf}}: \varvec{z} \mapsto P(\hat{\varvec{Y}}_{b})$ outputs the probability of an input bag’s class label. During the training of the model, the parameters $\theta _\mathrm{enc}$, $\theta _\mathrm{att}$, and $\theta _\mathrm{clf}$ for functions $f_\mathrm{enc}$, $f_\mathrm{att}$, and $f_\mathrm{clf}$ are optimized by minimizing the following problem:5where

6 $$\begin{aligned} P(\hat{\varvec{Y}}_b) = f_\mathrm{clf} \left( \sum _{i \in \mathcal I_b}f_\mathrm{att} \left( f_\mathrm{enc}(\varvec{x}_i)\right) f_\mathrm{enc}(\varvec{x}_i)\right) . \end{aligned}$$

In Eq. (5), ${\mathcal {L}}$ is the cross-entropy loss function for the bag class prediction.

**Fig. 2:**
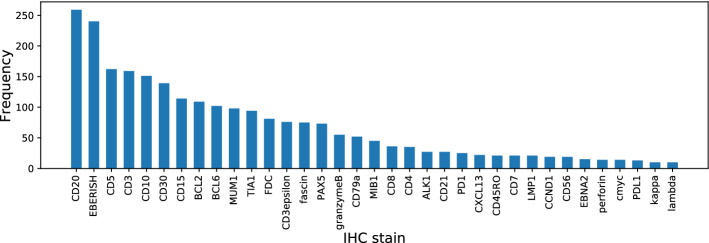
A histogram of the usage of each IHC stain, wherein we can observe that important IHC stains such as CD20 are used often

## Experiments

We first apply MDS to IHC staining patterns for embedding them to a low-dimensional space and typicalities of all the instances are then calculated. In the classification experiments, we select instances according to the calculated typicality, and the classification performances with several instance selections are compared.

### Experimental setup

*Database* Our database of malignant lymphomas comprises $N=262$ clinical cases, which include three subtypes: 67, 97, and 98 cases of AITL, DLBCL, and CHL, respectively. There are strictly two types of CHL—nodular sclerosis and mixed cellularity types—but they are regarded as one type in this study. The pathological tissue specimens used in our experiments consist of sections from both lymphatic tissues and extranodal organs; the former includes the lymph node, tonsil, and spleen, and the latter includes the stomach, colon, bone marrow, and skin. All the tissue slides were collected from over 80 different institutions for diagnostic consultation, and they had the definitive diagnoses and lists of the IHC stains used in the diagnosis of an expert hematopathologist.

*Calculation of typicality* In this work, we considered 35 types of IHC stains, which were used for at least 10 instances because the IHC stains that were hardly used in the diagnostic routine could result in noise in the typicality calculation. That is, the size of the IHC staining pattern ${\mathbb {S}}_n$ was set as $L=35$. A histogram indicating the frequency of each IHC stain is presented in Fig. 2. In the histogram, we can observe that important IHC stains such as CD20, EBERISH, CD5, and CD3 were often used for many instances in the diagnosis and that there were existing IHC stains that were rarely used as compared with CD20. As mentioned in the previous section, we employ MDS for the low-dimensional embedding method. IHC staining patterns are embedded into three-dimensional vectors in this study ($M=3$), and the typicality is calculated using the three-dimensional values $\varvec{u}_n$. In computing typicality, we experimentally adjust the bandwidth parameter *w* for the kernel density estimation such that the probability density of each subtype is distributed evenly between the low and high values. We manually adjusted *w* in this study, but it should be automatically adjusted for a much larger dataset.

**Fig. 3:**
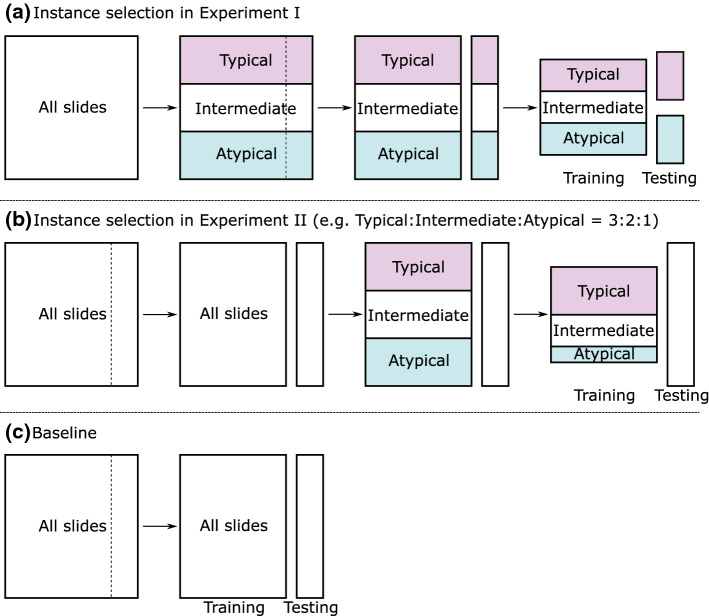
The instance selection method in our classification experiments. **a** Instance selection for testing data to validate the difficulty criteria of the classification using the same training data. **b** Instance selection for training data to confirm the improvement in generalization ability using typicality. **c** The baseline to be compared, that uses all the training data after the data splitting without any instance selection. Herein, the number of training data in (**c**) becomes larger than that in (**b**)

**Fig. 4:**
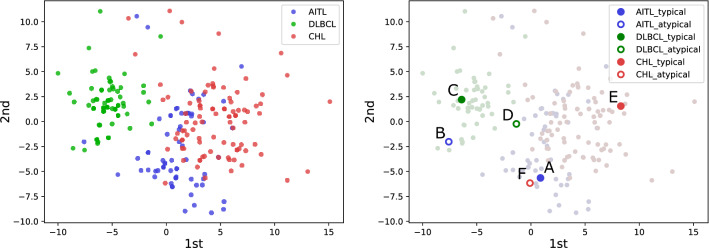
Left: The plots of low-dimensional IHC staining patterns embedded by MDS. The color of each dot indicates the subtype of each instance. We can observe that instances of the same subtype are clustered together, while the clusters of different subtypes are partially overlapped. The data in the overlapped regions have lower values of typicality. Right: The data corresponding to the instances listed in Table 1

*Subtype classification by MIL-CNN* We perform multi-class image classification, wherein each of the 262 slides is classified as AITL, DLBCL, or CHL. All the glass slides were digitized using a WSI scanner Aperio ScanScopeXT (Leica Biosystems, Germany) at 20x original magnification (0.50 $\mu$m/pixel). We used ResNet50 [27] as the feature extractor in $f_\mathrm{enc}$, and the output 2048-dimensional vector was reduced to a 512-dimensional vector $\varvec{h}$ $(Q=512)$ through the fully connected layer. In the experiments, in each training epoch, at most 5,000 image patches were randomly extracted from the tissue regions of a single WSI, and 50 bags were generated wherein each bag has 100 image patches.

Figure 3 illustrates the typicality-based instance selection methods in the classification experiments. In Experiment I, we investigate the relationship between the typicality computed from the IHC staining patterns and the difficulty of subtype identification based on the H&E-stained tissue images. Through this experiment, we aim to confirm that the typical instances are more classifiable when the classification model is trained with the same training dataset. All 262 slides are divided into three subsets ($K=3$) according to the labeled subtypes, and the data in each of the subsets are sorted according to the calculated typicality measures. We then split the data in each subset into three groups evenly according to typicality: typical, intermediately typical (called *intermediate*), and atypical groups. Each group is randomly divided into five subgroups, as shown in Fig. 3, and fivefold cross-validation is performed, wherein we compare the classification accuracy for the different testing data based on the group of typicality. All typical and atypical instances are used as testing data through fivefold cross-validation.

In Experiment II, we investigate whether we could improve the generalization ability of the subtype identification by changing the ratio of the number of data with different typicality in the training data. In contrast to Experiment I, we intend to confirm that the instance selection for the training slides based on their typicality improves the generalization ability of the subtype classification. In this experiment, first, all the dataset is randomly divided into five subgroups for fivefold cross-validation, as shown in Fig. 3. After calculating typicality and splitting all the data into three groups similarly to Experiment I, i.e., typical, intermediate, and atypical, we construct the training dataset by selecting data from each of the three groups. To obtain a superior training dataset, we vary the ratio of the number of data selected from each group under the condition that the resultant set should include data from all the groups. We experimentally determined that at least one-third of instances of each typicality group are used for the training dataset. For the baseline setting in this experiment, no instance selection is applied to the training data after the data splitting for fivefold cross-validation. All 262 instances are used as testing data through fivefold cross-validation in Experiment II.

In all the classification experiments, the training dataset is split into 75% training and 25% validation instances. During the testing, the trained models at epochs at which the model demonstrates the smallest losses for the validation data are employed.

**Table 1:** The examples of typical and atypical instances for each subtype

Instance	Subtype	Typicality	IHC stains
A	AITL	Typical	CD20, CD3, CD10, CD30, CD3epsilon,
			CD79a, CD8, CD4, CD56
B	AITL	Atypical	CD20, CD5, CD10, CD30, BCL2,
			CD15, BCL6, CD3epsilon, MIB1,CD79a,
			CCND1, CD7
C	DLBCL	Typical	CD20, CD5, CD3, CD10, BCL2, BCL6,
			MUM1, cmyc
D	DLBCL	Atypical	CD20, EBERISH, CD5, CD10, CD30,
			CD15, MUM1, PAX5
E	CHL	Typical	CD20, EBERISH, CD3, CD30, CD15,
			TIA1, FDC, fascin, PAX5,granzymeB,
			ALK1, perforin
F	CHL	Atypical	CD20, EBERISH, CD5, CD10, CD30,
			BCL2, CD15, TIA1, CD3epsilon,FDC,
			fascin, CD79a, CCND1, granzymeB

The first column “Instance” indicates the instance in Fig. 4. Generally, atypical instances have redundant IHC stains owing to the difficulty of selecting IHC stains in the observation of H&E-stained tissue images

### Results

#### Analysis of IHC staining pattern

We applied MDS to all 262 instances, and Fig. 4 presents the two-dimensional distribution of the embedded data plotted in the space spanned by the first and second components obtained via MDS, while all the embedded data comprise the three-dimensional vectors. It should be noted that subtype labels were not used in applying the MDS to the IHC staining patterns, while the instances of the same subtype were clustered together in the distribution of the embedded IHC stains. An embedded instance in a cluster would have a higher value of the typicality measure if the data is located far from the clusters of the other subtypes according to the definition of typicality in Eq. 3.

Table 1 lists examples of IHC staining patterns that have higher and lower typicality. The right panel of Fig. 4 presents the locations of the embedded data listed in Table 1. It can be observed that the IHC staining patterns were quite different even if the subtypes of those instances were the same. We found that atypical instances showed a tendency to have more IHC staining than typical instances. This is partly because more IHC stains were needed for the diagnosis of malignant lymphomas, as it was difficult to correctly predict the subtypes by referring to only the corresponding H&E-stained tissue images. Although the number of IHC stains for an atypical instance D is similar to that of a typical instance C, CD30, CD15, and PAX5 were used for the atypical instance. These IHC stains are usually used for identifying CHL, and it is assumed that an H&E-stained tissue slide of this DLBCL instance had a similar appearance to that of CHL. The distribution of the data embedded based on the distance of the IHC staining patterns changes depending on the difficulty of subtype identification based only on the H&E-stained tissue images.

**Table 2:** The comparison of the classification accuracy and macro-F1 score by fivefold cross-validation, wherein the testing data was selected based on typicality

Testing	Typical	Atypical
Accuracy	**0.698**	0.640
Macro-F1	**0.680**	0.618

Bold values in tables are the higher or highest evaluation measures in each setting

#### Classification with typicality-based instance selection

*Experiment I*

With the instance selection mentioned in the experimental setting, we performed subtype identification of AITL, DLBCL, and CHL using only H&E-stained tissue images. Table 2 lists the classification accuracy and macro-F1 score by fivefold cross-validation in Experiment I, wherein only typical or atypical slides were used for testing. As a result, the typical slides were identified more accurately than the atypical slides.

**Table 3:** The confusion matrices of the classification result with instance selection for the testing data, wherein the classification performance was improved in the case comprising the use of typical WSIs as the testing data

		Predict		
		AITL	DLBCL	CHL
*Testing data: Typical*				
	AITL	12	2	8
Correct	DLBCL	2	28	2
	CHL	7	5	20
*Testing data: Atypical*				
	AITL	10	6	6
Correct	DLBCL	3	26	3
	CHL	10	3	19

**Table 4:** The comparison of the classification accuracy and macro-F1 score in fivefold cross-validation, wherein all instances in the dataset were tested

Training	Baseline	Typical:Intermediate:Atypical					
		3:2:1	3:1:2	2:3:1	2:1:3	1:3:2	1:2:3
Accuracy	0.664	**0.683**	0.611	0.637	0.634	0.630	0.649
Macro-F1	0.648	**0.669**	0.596	0.619	0.615	0.609	0.637

Slides with different typicality were selected for the training data by changing the ratio of their number. “Typical:Intermediate:Atypical” denotes the ratio of the typical, intermediate, and atypical slides in the training data

Bold values in tables are the higher or highest evaluation measures in each setting

Table 3 shows the confusion matrices for both the classification results. It is thus revealed that the typical instances sampled based on typicality were more classifiable than the atypical ones, where recall was particularly improved for all the subtypes. Based on this experimental result, we can say that *typical* slides would have typical subtype-specific features in the images, while atypical slides have insufficient image features for subtype identification. It was confirmed that the typicality measure computed from the IHC staining patterns had a certain relationship with the difficulty of the subtype classification from H&E-stained WSIs.

*Experiment II* Next, the second instance selection presented in Fig. 3 was performed, wherein the slides for all typicalities were used for testing. Here, we changed the ratio of the number of data with different typicalities in the training data. In this experiment, we aimed to sample a superior training dataset that improves the generalization ability of the subtype identification even if the number of sampled data is reduced by varying the ratio of different typicalities included in the sampled data set. Table 4 lists the classification results obtained when the ratio of the instance selection was changed. Here, the instance selection “3:2:1” means that the resultant training dataset consists of all the data in the typical group, two-thirds of the data in the intermediate group, and one-third the data in the atypical group, where two-thirds and one-third of the slides were randomly sampled from the corresponding group, respectively. Table 4 shows that the classifier had the best accuracy when the training data was selected based on the typicality, especially when the training dataset included more typical instances and removed atypical instances. Note that the baseline method used a larger number of the training data than the method using instance selections. Although the method using instance selection had less training data than the baseline method, the method using the instance selection “3:2:1” achieved the best classification performance of all the settings.

**Fig. 5:**
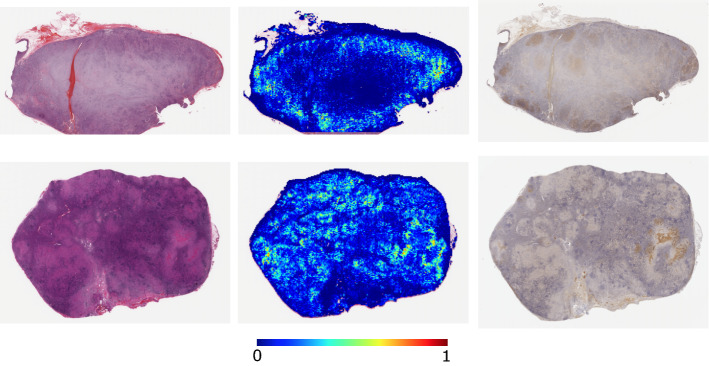
The visualization results of the attention weights as heat maps. For each instance, an H&E-stained tissue image, a visualized attention weight, and a CD30 IHC-stained tissue image are presented, wherein the highly attention-weighted regions in the heat map correspond to the positive regions in the CD30 IHC-stained tissue image

**Table 5:** The confusion matrices of the classification result with instance selection for the training data, wherein the classification performance was improved in the case of the focus on typical WSIs in the instance selection of the training data

		Predict		
		AITL	DLBCL	CHL
*Typical:Intermediate:Atypical = 3:2:1*				
	AITL	38	8	21
Correct	DLBCL	7	81	9
	CHL	25	13	60
*Typical:Intermediate:Atypical = 1:2:3*				
	AITL	35	6	26
Correct	DLBCL	8	74	15
	CHL	25	12	61

Table 5 lists the confusion matrices for two results of ratio “3:2:1” and “1:2:3,” which are the settings that focus on typical and atypical instances. In the comparison of these two confusion matrices, the classification performance for the DLBCL was found to be significantly improved. This result could be obtained because atypical DLBCL instances, that had the similar appearance of H&E-stained tissue slides to CHL instances as mentioned in Sect. 4.2.1, were removed from the training data by the proposed instance selection. Using typicality as a criterion for the instance selection to construct the training dataset, we can improve the generalization ability of the subtype classifier. As the typicality can be calculated from only the IHC staining patterns that are obtained from medical records, the typicality measure would help construct appropriate training data for better subtype classifiers.

*Visualization of attention weight* We visualized the attention weights computed by our best classifier in Experiment II. The validity of attention-based MIL for DLBCL has already been confirmed in the literature [24]. Figure 5 presents the visualization results of attention weights for CHL. The images in the middle column present the visualized attention weights as heat maps, wherein the attention weights were normalized between 0 and 1 in each bag and blue-to-red heat maps were generated. The right column presents the CD30 IHC stained tissue images of serial sections of the same patients. The CD30 antibody is often used for CHL tissue specimens and reacts positively. We can observe that the brown regions in the CD30-stained tissue images have higher attention weights in the corresponding regions of heat maps, and it was confirmed that the attention network could focus on the important regions in the entire tissue slides.

## Conclusion

In this paper, we proposed a method for improving the generalization ability of a classification model that classifies the subtypes from H&E-stained WSIs of malignant lymphomas. We defined the typicality of an H&E-stained WSI from a set of IHC stains described in a medical record that indicates how typical its appearance is. The proposed typicality can be employed as a criterion in the instance selection to improve the generalization ability of the subtype classification. We performed subtype classification of malignant lymphomas wherein datasets were selected according to the computed typicality in order to investigate the relationship between the typicality calculated from the IHC staining pattern and the subtype classification difficulty and improve the generalization ability. The results confirmed that the typicality of subtypes was related to the classification performance, and we showed that instance selection based on typicality would be useful for better construction of the subtype classifier. By considering the typicality of the case, we constructed an appropriate dataset for realizing a higher classification performance with the small number of training data as compared to the baseline. For the application of the proposed typicality, instance selection on a large dataset is considered for reducing costs for the scanning of WSIs and the training of models. Another application is the instance selection for class-imbalance dataset such that large-class instances are sampled to adjust to the small class, wherein random sampling can degrade the model performance by sampling atypical instances. The proposed typicality, which is calculated from a set of IHC stains, can be used as a measure in various instance selections.
